# Pulp-to-palm distance after plate fixation of a distal radius fracture corresponds to functional outcome

**DOI:** 10.1186/s40945-023-00159-4

**Published:** 2023-03-20

**Authors:** Hugo Jakobsson, Eva Lundqvist, Per Wretenberg, Marcus Sagerfors

**Affiliations:** Department of Hand and Orthopedic Surgery, Faculty of Medicine and Health, Örebro University, SE 70182 Örebro, Sweden

**Keywords:** Distal radius fracture, Outcome, Pulp-to-palm distance, Wrist function, Rehabilitation, Patient-related outcome measures, Plating

## Abstract

**Introduction:**

Several factors can influence the outcome after a distal radius fracture (DRF). The aim of this study was to assess whether postoperative pulp-to-palm (PTP) distance correlated with functional outcomes after plate fixation of DRF.

**Materials & methods:**

This is a secondary analysis of a randomized controlled trial aimed to investigate the effects of plate fixation in patients with type-C fractures. Subjects (*N* = 135) were divided into 2 groups based on PTP distance (equal to or higher than 0 cm) at 4 weeks postoperatively. Outcome measures were collected prospectively at 3, 6 and 12 months and included Patient-Rated Wrist Evaluation (PRWE), Quick Disabilities of the Arm Shoulder and Hand (QuickDASH) scores, wrist range of motion (ROM), Visual Analog Scale (VAS) pain scores, and hand grip strength.

**Results:**

Overall, at 3 and 6 months patients with PTP > 0 cm had significantly worse outcomes (PRWE, QuickDASH, wrist ROM) than those with PTP =0 cm. At 12 months, QuickDASH and wrist ROM were still significantly worse. In the volar-plating subgroup, patients with PTP > 0 cm had significantly worse wrist ROM and grip strength at 3 months, but no significant differences were found in subsequent follow-ups. In the combined-plating group, patients with PTP > 0 cm had significantly worse QuickDASH, wrist ROM and grip strength at 3 months. At 6 and 12 months, wrist ROM was still significantly worse.

**Conclusions:**

Measurement of PTP distance appears to be useful to identify patients likely to have worse outcome after plating of a DRF. This could be a tool to improve the allocation of hand rehabilitation resources.

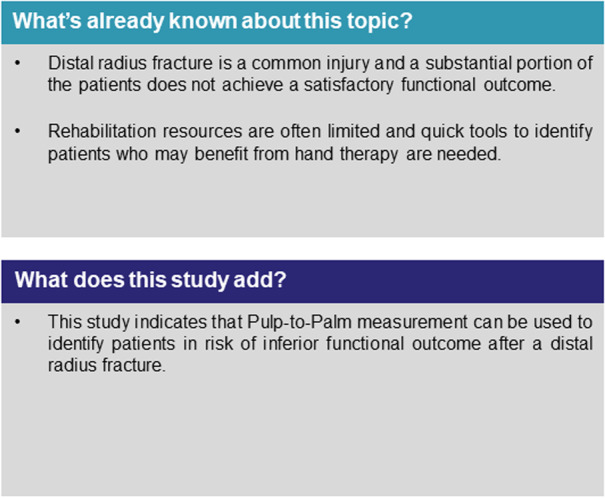



## Introduction

The distal radius fracture (DRF) is the most common fracture in adults, representing 18% of all fractures in an orthopedic trauma unit [1]. A majority of the fractures can be managed non-operatively, but unstable or non-reducible fractures are often considered candidates for surgical treatment [2]. Treatment depends on patient age and activity level, fracture type, surgeon and patient preference [3]. Plate fixation is the most common treatment modality. Both volar locking plates and combined dorsal and volar plate fixation have demonstrated good outcomes [4–6]. Most patients recover well from a DRF, but 17% report major disability at 1-year follow-up, and more than half of these patients continue to report major disability at intermediate follow-up [7].

The need for physical therapy after a distal radius fracture is debated. Some authors have found improved wrist range of motion (ROM) after supervised hand therapy [8, 9], but other reports favor home exercise programs [10, 11]. A systematic review found insufficient evidence from randomized controlled trials to support a home program or therapist-supervised clinic-based program following a DRF without complications [12]. Valdes et al. have concluded that some patients may benefit from supervised hand therapy [13].

Several factors are known to influence the outcome after a DRF, including injury compensation, education, other medical comorbidities [14], self-efficacy [15], hand grip strength and dominant hand injury [16], baseline pain intensity [17], as well as age and income [18]. In the clinical setting, rehabilitation resources are often limited. Tools to identify patients at risk for an inferior outcome could potentially improve the allocation of rehabilitation resources.

The validity of pulp-to-palm distance (PTP) as a measure of finger flexion has been established by MacDermid [19]. A previous study indicated that a PTP distance > 0 cm was associated with an inferior functional outcome 3 months after combined plating of a DRF [20]. It is unknown whether this association persists after 3 months postoperatively and whether it is seen after volar plating of a DRF.

The aim of this study was to investigate if patients with PTP distance > 0 cm postoperatively have worse hand function up to 1 year after surgery of a DRF compared to patients with PTP distance equal to 0 cm.

## Methods

### Participants

This is a secondary analysis of a randomized controlled trial (RCT) aimed to investigate the effects of volar versus combined plate fixation in 150 patients with type C fractures [21]. The study showed similar results between the treatment groups, but a higher incidence of adverse events in the groups treated with combined plating. The study participants were enrolled between June 15, 2017 and July 31, 2019. Patients with a DRF referred to the Department of Hand Surgery at Örebro University Hospital in Sweden were eligible for the study. The department is a tertiary referral center for complex DRFs. The inclusion criteria were unstable intra-articular DRF AO type C [22] with an articular displacement of > 2 mm or a dorsal angulation of > 20 degrees, age 18-80. The exclusion criteria were dementia, substance abuse, impaired autonomy, inability to comprehend the Swedish language, fractures older than 12 days, previous DRF of either wrist, concomitant carpal ligament injury and combined fracture of the ipsilateral arm or hand. Patients that met the inclusion criteria and consented to participate in the study were randomized to surgical treatment with either a volar locking plate or combined volar and dorsal plating. Randomization was performed by an operating room nurse randomly drawing an opaque sealed envelope containing treatment modality. There were 75 envelopes containing each treatment modality. If the surgeon established during surgery that the inclusion criteria were not met, the patient was excluded from the study and a new envelope was added.

### Ethical considerations

The study was approved by the Swedish ethical review authority (reference number 2016/455 and 2021-06576-02) and registered in Swedish research database https://www.researchweb.org/is/sverige (reference number 274674), retrospectively registered December 5, 2019.

### Surgical technique

All procedures were performed by specialists in hand surgery (experience level 3 and 4 according to Tang and Giddins) [23] at the Department of Hand Surgery,Örebro University Hospital, using the same approach and technique. The surgery was performed in general anesthesia and brachial plexus block. The implants used were manufactured by Trimed Inc. (Santa Clarita, CA, USA). The plates used for combined plating were developed by our unit in cooperation with Trimed Inc.

**Fig. 1:**
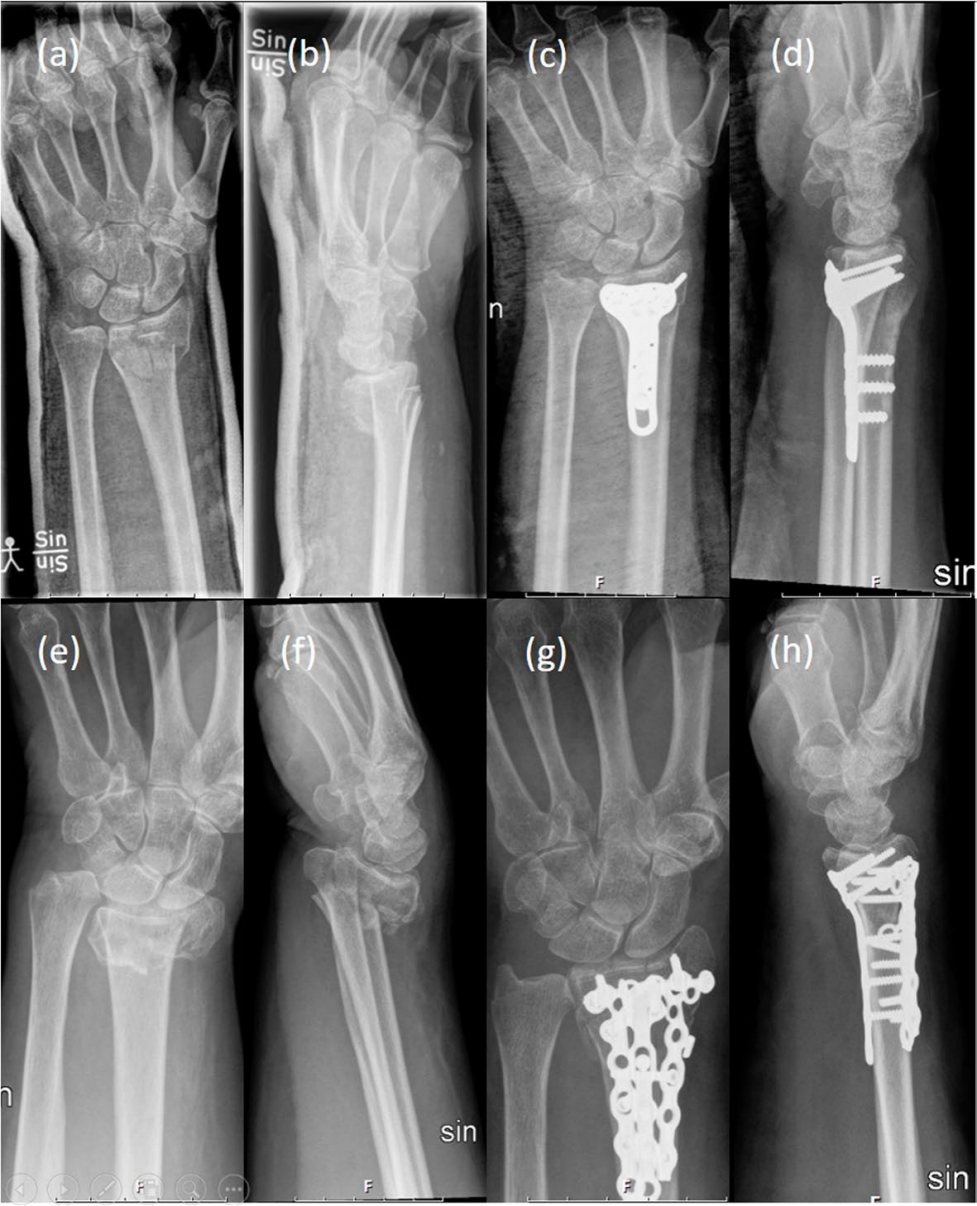
Distal radius fracture reduced and fixated with volar plating (**a-d**) and combined plating (**e-h**)

For the volar plate fixation, a volar central approach was used. The volar incision was made centrally, releasing the carpal tunnel, approaching the fracture in the interval between the median nerve/flexor pollicis longus tendon and the flexors of the fingers. The fracture was reduced and fixated with a volar locking plate. If needed, the brachioradialis tendon insertion was released. The pronator quadratus was repaired using absorbable sutures if feasible (Fig. 1a-d).

The combined plating was performed by approaching the fracture volarly as described above and fixating the volar locking plate. Next, a straight skin incision over the 4th dorsal compartment was made. The extensor retinaculum was incised z-shaped through the 4th compartment and the fracture was exposed. A Trimed dorsal plate was placed and fixated proximally, pushing the fragments in place distally with the dorsal plate and using the volar plate as a fulcrum. Distally, locking screws were used (Fig. 1e-h).

### Postoperative regimen

The day after surgery the patients were given instructions regarding edema control, finger and shoulder movement and pain reduction by a hand therapist before discharge from the hospital. The wrist was immobilized in a volar cast for 2 weeks. Two weeks postoperatively, the patients were followed up by a hand surgeon, and a wrist X-ray was performed. The patients started physiotherapy under the guidance of a hand therapist and were given a prefabricated removable wrist orthosis. The orthosis was to be used for 2 weeks and only removed during range of motion exercises. Continued use of the orthosis was decided by the patient. The patients were instructed to use the injured hand in light daily activities. The patients received instructions to exercise the wrist for 10 minutes 4-5 times a day at home and to refrain from heavy loading until 12 weeks postoperatively. Only active mobilization exercises were used. The program continued for 3 months. The patients who had not achieved a satisfactory result at this time continued the program with passive exercises added. The patients met with a hand therapist for follow-up at 2 and 4 weeks, 3, 6 and 12 months after surgery. Further visits with a hand surgeon with X-ray of the wrist were completed 3 and 12 months postoperatively.

### Outcome and measurements

The PTP distance was assessed according to the description by Macey et al. and MacDermid et al. [19, 24] using a ruler to measure the direct distance between the distal palmar crease and the nail to distal nail fold junction. The value of the finger with the longest distance was used. Baseline PTP distance was not registered, but there were no signs of old finger injuries or congenital malformations limiting the ability to make a full fist preoperatively.

The hand function was assessed using the following patient-reported outcome measures (PROMs): Patient-Rated Wrist Evaluation score (PRWE) [25] and Quick Disabilities of the Arm, Shoulder and Hand score (QuickDASH) [26]. The PRWE is a 15-item questionnaire rating pain and disability in functional activities, and the psychometric properties are well described [25, 27]. A score from 0 to 100 is calculated with zero representing no pain or disability. The QuickDASH is an 11-item questionnaire measuring physical function and symptoms in people with musculoskeletal disorders of the upper limb. A score is calculated and ranges from zero (no disability) to 100 (most severe disability) [26]. The PRWE and QuickDASH assessments were made using the validated Swedish translations [26, 28]. Additional outcomes included pain measured with the Visual Analog Scale (VAS) at rest and during activity (0 cm = no pain, 10 cm = worst imaginable pain), wrist ROM (extension, flexion, pronation, supination, ulnar and radial deviation), and grip strength. All measurements were recorded by an experienced hand therapist, in accordance with the guidelines from the Swedish national quality registry for hand surgery [24]. Wrist ROM and grip strength measurements were performed by a hand therapist using a goniometer and a calibrated hand dynamometer (E-link Hand Kir, Biometrics Ltd., Newport UK.), respectively. Measurements were done with the patient seated and the forearm in neutral rotation. Three attempts were made on both sides, starting with the unaffected side, and a mean value was recorded.

At 4 weeks postoperatively, PTP distance, VAS at rest and during activity and ROM were recorded. At 3, 6, and 12 months, PRWE, QuickDASH, VAS at rest and during activity, wrist ROM and grip strength were recorded. At 12 months, wrist ROM and grip strength of the uninjured hand were also recorded. The wrist ROM and grip strength at 3 months was divided by the values of the uninjured hand at 12 months in order to calculate the values as percentage of the uninjured hand.

### Statistical analysis

In the primary study, at least 63 patients in each group was deemed necessary to detect a difference of 10 points in the QuickDASH score with a statistical power of 80% and an α-level of 0.05 [21]. Post hoc power analyses were performed. The sample size was the participants eligible at the 12 months follow up. The α-level was set to 0.05. The effect sizes used in the analyses were 0.8, 0.5 and 0.2.

IBM SPSS Statistics Version 25.0 was used for statistical analysis. Normally distributed data were presented as mean and standard deviation. Non-normally distributed data were presented as median and interquartile range. Distribution of data was tested with Shapiro-Wilks test. The entire cohort, as well as two subgroups (volar and combined plating), were each divided into two groups: PTP distance at 4 weeks postoperatively =0 cm and > 0 cm. Comparison between groups (PTP distance equal or higher than 0 cm) regarding PRWE, QuickDASH, pain, wrist ROM and grip strength at 3, 6 and 12 months was performed using Mann-Whitney U-test. A *p*-value < 0.05 was considered significant.

## Results

**Fig. 2:**
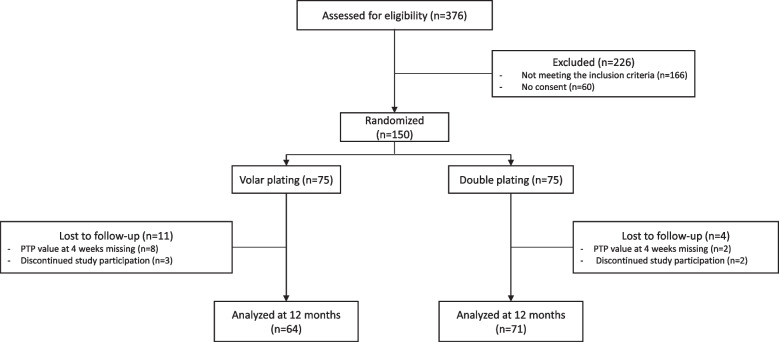
Details of patient inclusion

During the period June 15, 2017 to July 31, 2019, 376 patients were treated surgically for DRF at the Department of Hand Surgery at Örebro University Hospital. Of these patients, 150 were included in the RCT and randomized to volar or combined plating. At 4 weeks, PTP distance was missing in 10 patients, thus excluded. At 3 and 6 month, 140 patients were eligible for analysis. At 12 months, 5 patients withdrew from the study. Figure 2 shows details for patient inclusion.

In the volar plating group, 11 patients had a PTP distance > 0 cm and 56 patients 0 cm at 4 weeks postoperatively. In the combined plating group, 25 patients had PTP distance > 0 cm and 48 patients 0 cm at 4 weeks postoperatively. Baseline data are presented in Table 1.

**Table 1:** Baseline characteristics

	Combined plating		Volar plating		Combined and volar plating	
	Pulp-to-palm distance = 0 cm	Pulp-to-palm distance > 0 cm	Pulp-to-palm distance = 0 cm	Pulp-to-palm distance > 0 cm	Pulp-to-palm distance = 0 cm	Pulp-to-palm distance > 0 cm
Number of patients	48	25	56	11	104	36
Age (years) median (IQR)	60 (50-71)	63 (58-71)	60 (50-70)	58 (49-63)	60 (50-71)	63 (55-69)
Female sex n (%)	39 (81.3)	17 (68)	44 (78.6)	9 (81.8)	83 (79.8)	26 (72.2)
Fracture type AO C.1 n (%)	15 (31.3)	4 (16)	13 (23.2)	3 (27.3)	28 (26.9)	7 (19.4)
Fracture type AO C.2 n (%)	18 (37.5)	9 (36)	25 (44.6)	2 (18.2)	38 (36.5)	11 (30.6)
Fracture type AO C.3 n (%)	15 (31.3)	12 (48)	18 (32.1)	6 (54.5)	33 (31.7)	18 (50)
Operating time (minutes) mean (SD)	83.7 (15.2)	94 (20.2)	59.8 (22.0)	62.6 (26.1)	70.8 (22.5)	84.4 (26.2)

Age is presented as median (interquartile range). Female sex and fracture types are presented as n. Operating time is presented as mean (SD)

*AO* Arbetsgemeinschaft für Osteosynthesefragen

The post hoc power analyses showed an achieved power of 0.97 for the comparison between the groups with and without PTP distance for an effect size of 0.8; an achieved power of 0.68 for an effect size of 0.5; and an achieved power of 0.16 for an effect size of 0.2.

### Volar and combined plating

At 3 months all outcome measures, except VAS pain scores at rest and during activity, were significantly worse in the PTP > 0 cm group (Table 2). At 6 months all outcome measures were significantly worse in the PTP > 0 cm group, except PRWE score and both VAS pain scores (Table 3). At 12 months follow up the PTP > 0 cm group had significantly worse QuickDASH score, wrist extension, flexion and pronation (Table 4).

**Table 2:** Wrist function 3 months after surgery (*N* = 140)

	Combined plating			Volar plating			Combined and volar plating		
	PTP distance = 0 cm	PTP distance > 0 cm	***P***-value	PTP distance = 0 cm	PTP distance > 0 cm	***P***-value	PTP distance = 0 cm	PTP distance > 0 cm	***P***-value
PRWE median (IQR)	24 (14.5-35.5)	29.5 (19-50)	0.12	14.5 (10-28.5)	25 (9.5-47)	0.26	21 (11-31)	26 (18-49)	**0.013**
QuickDASH median (IQR)	25 (15.9-31.8)	34.1 (22.7-54.5)	**0.006**	15.9 (6.8-29.5)	18.2 (11.4-40.9)	0.43	21 (11-31)	30 (20-51)	**0.002**
VAS at rest median (IQR)	0 (0-0)	0 (0-1.4)	0.10	0 (0-1)	0 (0-0)	0.57	0 (0-1)	0 (0-1)	0.29
VAS during activity median (IQR)	3 (2-4)	3 (2-5)	0.23	2 (0-3)	1 (0-3.5)	0.65	2 (1-4)	3 (2-5)	0.19
Extension median (IQR)	40° (31°-45°)	25° (15°-39°)	**< 0.001**	50° (40°-60°)	35° (30°-45°)	**0.005**	45° (36°-55°)	30° (20°-40°)	**< 0.001**
Flexion median (IQR)	45° (40°-55°)	38° (30°-45°)	**0.001**	55° (50°-65°)	50° (45°-55°)	**0.045**	55° (45°-60°)	45° (35°-50°)	**< 0.001**
Pronation median (IQR)	75° (70°-80°)	75° (65°-79°)	0.25	75° (70°-80°)	75° (70°-80°)	0.61	75° (70°-80°)	75° (65°-80°)	0.12
Supination median (IQR)	75° (65°-80°)	65° (46°-75°)	**0.014**	73° (70°-80°)	70° (50°-75°)	0.14	75° (65°-80°)	70° (50°-75°)	**0.002**
Radial deviation median (IQR)	15° (10°-20°)	10° (5°-10°)	**< 0.001**	20° (15°-20°)	20° (15°-20°)	0.58	15° (15°-20°)	10° (10°-15°)	**< 0.001**
Ulnar deviation median (IQR)	25° (21°-30°)	20° (15°-24°)	**< 0.001**	30° (25°-35°)	30° (25°-30°)	0.13	30° (25°-35°)	20° (20°-25°)	**< 0.001**
Grip strength kilograms median (IQR)	15 (12-19)	12 (8-16)	**0.006**	18 (14-22)	14 (11-16)	**0.019**	16 (13-21)	12 (10-16)	**< 0.001**
Extension %^a^ median (IQR)	64 (51-74)	38 (21-57)	**0.002**	75 (61-86)	56 (46-72)	**0.011**	69 (56-80)	46 (32-64)	**< 0.001**
Flexion %^a^ median (IQR)	63 (52-75)	53 (46-64)	**0.022**	75 (64-88)	73 (63-83)	0.87	69 (60-81)	63 (50-71)	**0.009**
Pronation %^a^ median (IQR)	94 (81-100)	89 (75-100)	0.64	94 (88-100)	91 (78-96)	0.51	94 (83-100)	89 (76-100)	0.40
Supination %^a^ median (IQR)	88 (82-94)	78 (57-89)	**0.027**	88 (82-100)	83 (61-90)	0.15	88 (82-94)	78 (62-89)	**0.006**
Radial deviation %^a^ median (IQR)	75 (50-100)	50 (33-67)	**0.001**	78 (67-100)	78 (64-100)	0.90	75 (58-100)	50 (33-78)	**0.002**
Ulnar deviation %^a^ median (IQR)	75 (57-86)	57 (43-75)	**0.015**	78 (67-100)	71 (62-86)	0.21	75 (63-88)	63 (50-82)	**0.003**
Grip strength %^a^ median (IQR)	61 (48-69)	45 (27-58)	**0.001**	65 (54-80)	51 (47-70)	0.12	63 (50-76)	48 (35-60)	**< 0.001**

The values are presented as median (interquartile range). Comparison between groups were made using Mann-Whitney U test

*VAS* Visual Analog Scale, *PRWE* Patient Rated Wrist Evaluation, *QuickDASH* Quick Disabilities of the Arm Shoulder and Hand

^a^measure expressed as percentage of the uninjured wrist

**Table 3:** Wrist function 6 months after surgery (*N* = 140)

	Combined plating			Volar plating			Combined and volar plating		
	PTP distance = 0 cm	PTP distance > 0 cm	***P***-value	PTP distance = 0 cm	PTP distance > 0 cm	***P***-value	PTP distance = 0 cm	PTP distance > 0 cm	***P***-value
PRWE median (IQR)	19.5 (6.6-33.1)	21 (10-44)	0.44	10.3 (1-21.9)	12 (5.3-23.8)	0.47	13 (5-25)	19 (7-40)	0.103
QuickDASH median (IQR)	13.6 (6.2-28.4)	22.7 (9.1-36.4)	0.13	6.8 (2.3-15.3)	13.7 (1.7-23.9)	0.41	10 (4-21)	16 (9-36)	**0.013**
VAS at rest median (IQR)	0 (0-0)	0 (0-2)	0.12	0 (0-0.8)	0 (0-0)	0.10	0 (0-0)	0 (0-1)	0.591
VAS during activity median (IQR)	2.8 (0-5)	2.8 (2-4.3)	0.89	1 (0-3)	1 (0-3.5)	0.95	2 (0-3.25)	2.5 (1-4)	0.372
Extension median (IQR)	45° (35°-51°)	35° (20°-45°)	**0.002**	55° (50°-60°)	53° (44°-70°)	0.75	50° (44°-60°)	40° (25°-53°)	**0.001**
Flexion median (IQR)	50° (45°-61°)	40° (35°-50°)	**0.002**	65° (60°-75°)	68° (60°-70°)	1	60° (50°-70°)	50° (35°-65°)	**0.002**
Pronation median (IQR)	75° (70°-85°)	70° (65°-80°)	0.16	80° (75°-85°)	78° (68°-90°)	0.46	80° (70°-85°)	75° (65°-80°)	**0.027**
Supination median (IQR)	75° (69°-85°)	65° (50°-75°)	**0.009**	80° (70°-85°)	83° (73°-86°)	0.52	78° (70°-85°)	75° (60°-80°)	**0.021**
Radial deviation median (IQR)	20° (15°-20°)	15° (10°-15°)	**0.011**	20° (16°-25°)	20° (15°-25°)	0.44	20 °(15°-25°)	15° (10°-20°)	**0.003**
Ulnar deviation median (IQR)	25° (20°-30°)	20° (20°-30°)	0.14	35° (26°-40°)	30° (29°-35°)	0.46	30° (25°-35°)	30° (20°-30°)	**0.033**
Grip strength kilograms median (IQR)	19 (15-24)	16 (13-19)	0.11	21 (16-26)	20 (17-23)	0.33	20 (15-25)	17 (13-21)	**0.024**

The values are presented as median (interquartile range). Comparison between groups were made using Mann-Whitney U test

*VAS* Visual Analog Scale, *PRWE* Patient Rated Wrist Evaluation, *QuickDASH* Quick Disabilities of the Arm Shoulder and Hand

**Table 4:** Wrist function 12 months after surgery (*N* = 135)

	Combined plating			Volar plating			Combined and volar plating		
	PTP distance = 0 cm	PTP distance > 0 cm	***P***-value	PTP distance = 0 cm	PTP distance > 0 cm	***P***-value	PTP distance = 0 cm	PTP distance > 0 cm	***P***-value
PRWE median (IQR)	11.5 (3.6-26.6)	20.5 (3-34)	0.53	3.5 (0-9)	8.8 (0-17.6)	0.49	5 (1-15)	12 (3-27)	0.089
QuickDASH median (IQR)	9.2 (2.3-23.3)	13.6 (4.5-36.4)	0.17	4.5 (0-7.4)	5.7 (0-21)	0.45	7(2-14)	14 (2-33)	**0.020**
VAS at rest median (IQR)	0 (0-0)	0 (0-1)	0.53	0 (0-0)	0 (0-0)	0.32	0 (0-0)	0 (0-0)	0.618
VAS during activity median (IQR)	1.3 (0-3)	2 (0-4)	0.34	0 (0-1)	0.3 (0-2.3)	0.49	0.5 (0-2)	1 (0-3)	0.098
Extension median (IQR)	50° (45°-55°)	35° (25°-50°)	**< 0.001**	60° (50°-70°)	55° (44°-61°)	0.28	55° (48°-60°)	45° (30°-50°)	**< 0.001**
Flexion median (IQR)	60° (50°-65°)	50° (35°-55°)	**< 0.001**	70° (65°-75°)	68° (63°-70°)	0.16	65° (60°-70°)	50° (40°-65°)	**< 0.001**
Pronation median (IQR)	80° (71°-90°)	80° (75°-90°)	0.73	80° (78°-90°)	80° (74°-86°)	0.46	80° (75°-90°)	80° (75°-88°)	0.693
Supination median (IQR)	80° (70°-90°)	80° (60°-80°)	0.15	80° (70°-90°)	78° (68°-86°)	0.32	80° (70°-90°)	80° (65°-80°)	0.051
Radial deviation median (IQR)	20° (15°-20°)	15° (10°-20°)	0.055	20° (20°-25°)	20° (19°-21°)	0.65	20° (15°-23°)	15° (15°-20°)	**0.019**
Ulnar deviation median (IQR)	30° (25°-35°)	30° (20°-35°)	0.41	35° (30°-40°)	35° (30°-40°)	0.67	30° (28°-40°)	30° (25°-40°)	0.678
Grip strength kilograms median (IQR)	22 (16-28)	19 (17-24)	0.30	23 (19-27)	21 (19-25)	0.42	22 (18-27)	20 (18-24)	0.094

The values are presented as median (interquartile range). Comparison between groups were made using Mann-Whitney U test

*VAS* Visual Analog Scale, *PRWE* Patient Rated Wrist Evaluation, *QuickDASH* Quick Disabilities of the Arm Shoulder and Hand

### Volar plating

At 3 months postoperatively, the patients with PTP > 0 cm had significantly inferior wrist extension, flexion and grip strength (Table 2). At 6 and 12 months after surgery, no significant differences between the two groups were found (Tables 3 and 4).

### Combined plating

Three months postoperatively, there was a significantly worse QuickDASH score, wrist extension, flexion, supination, radial deviation, ulnar deviation and grip strength in the group with PTP distance > 0 cm (Table 2). At 6 months after surgery, this group had significantly worse wrist extension, flexion, supination and radial deviation (Table 3). After 12 months, wrist extension and flexion were still significantly worse (Table 4).

## Discussion

The aim of this study was to investigate if patients with postoperative PTP distance > 0 cm have worse clinical outcome after a DRF. The study is based on a RCT with a relatively large group of patients. The treatment was uniform, with surgeries performed by fellowship-trained orthopedic hand surgeons, and all patients had the same amount of hand therapy.

PTP distance has previously been associated to finger flexion measures [19], but studies on the role of PTP distance as a predictor of outcome after DRF are scarce. Valdes et al. found that decreased finger flexion was an important predictor for ROM and PRWE score 6 months postoperatively in a study of 50 patients with DRF treated with volar plate fixation [13].

Another study assessing combined plating found that patients with a DRF who underwent surgery with combined plating and had a PTP distance > 0 cm were more likely to have worse wrist function at 3 months after surgery [20]. We found that patients with PTP distance > 0 cm have significantly worse patient-reported and clinical outcomes after surgery with either volar or combined plating at 3 and 6 months postoperatively. In the group treated with combined plating, the patients with PTP distance > 0 cm 4 weeks postoperatively had significantly worse QuickDASH scores, ROM and grip strength 3 months postoperatively. Although QuickDASH scores differed significantly, the threshold for the minimal clinically important difference (MCID) was not reached [29].

At 6 months they had significantly worse wrist ROM. The unfavorable outcome in the whole cohort and the patients treated with combined plating with PTP > 0 cm group was not as pronounced in patients that underwent surgery with volar plating. These patients had significantly worse wrist extension, flexion and grip strength 3 months after surgery, but no significant difference at 6 and 12 months after surgery. Hence, the findings of our study are in line with previous studies.

The VAS pain scores were low and similar in both the volar plate and combined plating groups. This is encouraging, as pain has been reported as the most important factor after wrist surgery [30]. Pain at rest and movement-evoked pain have also been suggested as tools to predict outcome after a DRF [31]. A combination of pain and PTP distance might enhance the specificity when screening for patients with potentially bad outcomes after DRF surgery. Future studies assessing this relationship would be interesting.

Wrist ROM is arguably an important outcome measure for wrist function, and a direct correlation between wrist ROM and patient-reported outcome measures has been reported [32]. The operating time was substantially longer for the combined plating. This, as well as the combination of volar and dorsal incisions, are likely to impact scar formation around the wrist and consequently ROM. A previous study [33] found that combined plating is associated with inferior wrist ROM compared to volar plating, and our study concurs.

The patients with a PTP distance > 0 cm with either volar or combined plating had significantly inferior grip strength up to 3 months postoperatively. For the volar plate group, the patients with a PTP distance =0 cm had a grip strength of 57% of the uninjured hand 3 months postoperatively, which is somewhat lower compared to the findings by Landgren et al., who found a grip strength injured/uninjured ratio of 69% [34]. However, the two populations are not entirely comparable since the patients in that study were treated with a volar plate or fragment-specific fixation using a Henry approach. In addition, our patients had restrictions on load-bearing in the first 10-12 weeks, which may have had an impact on grip strength. A recent study found that a weak grip strength compared to the uninjured side was associated with a worse QuickDASH score 12 months after volar plating of a DRF [16]. Interventions aiming at improving short-term outcome for targeted patients could potentially focus on grip strength. This may lead to decreased sick leave after DRF surgery, but further studies are needed to assess this issue and what interventions are optimal.

Dewan et al. found that the majority of improvement in fracture-specific pain/disability was completed at 6 months after the fracture [35]. In contrast, another study found that 16% of individuals had ongoing pain and disability at 1 year after a DRF [36]. The patients in this study were all treated operatively for a complex intraarticular fracture and the outcome measures improved until 12 months after surgery, which may suggest that the rehabilitation process can take up to 1 year.

### Limitations

Surgery with volar plating resulted in fewer patients with PTP distance > 0 cm (*n* = 11), compared to combined plating (*n* = 25). As a consequence, the statistical analysis has less power. A larger cohort would likely have been able to catch differences in more of the studied outcome measures.

## Conclusions

In conclusion, patients with PTP distance > 0 cm after DRF surgery have worse outcomes up to 1 year after volar and combined plating. Our study suggests that PTP distance 4 weeks postoperatively may be used as a screening tool of worse clinical outcomes in patients with DRFs treated with volar or combined plating. It is quick and easy to use in the clinical setting. Future studies are needed to investigate whether intensified supervised therapy can improve outcomes in these patients and what interventions are optimal.

## Data Availability

The datasets used in this study are available from the corresponding author on reasonable request.

## Abbreviations

AO: Arbetsgemeinschaft für Osteosynthesefragen
DRF: Distal Radius Fracture
MCID: Minimal Clinically Important Difference
PROMs: Patient-Reported Outcome Measures
PRWE: Patient-Reported Wrist Evaluation
PTP: Pulp-to-palm
QuickDASH: Quick Disabilities of the Arm, Shoulder and Hand
RCT: Randomized Controlled Trial
ROM: Range of Motion
VAS: Visual Analog Scale
